# NAD Modulates DNA Methylation and Cell Differentiation

**DOI:** 10.3390/cells10112986

**Published:** 2021-11-02

**Authors:** Simone Ummarino, Clinton Hausman, Giulia Gaggi, Lucrezia Rinaldi, Mahmoud A. Bassal, Yanzhou Zhang, Andy Joe Seelam, Ikei S. Kobayashi, Marta Borchiellini, Alexander K. Ebralidze, Barbara Ghinassi, Bon Q. Trinh, Susumu S. Kobayashi, Annalisa Di Ruscio

**Affiliations:** 1 Harvard Medical School Initiative for RNA Medicine, Harvard Medical School, Boston, MA 02115, USA; mbassal@bidmc.harvard.edu (M.A.B.); yzhang16@bidmc.harvard.edu (Y.Z.); aebralid@bidmc.harvard.edu (A.K.E.); btrinh@bidmc.harvard.edu (B.Q.T.); 2 Harvard Stem Cell Institute, Harvard Medical School, Boston, MA 02115, USA; skobayas@bidmc.harvard.edu; 3 Department of Medicine and Cancer Research Institute, Beth Israel Deaconess Medical Center, Harvard Medical School, Boston, MA 02215, USA; chausman@bidmc.harvard.edu (C.H.); ggaggi@bidmc.harvard.edu (G.G.); lrinald1@bidmc.harvard.edu (L.R.); ikobayas@bidmc.harvard.edu (I.S.K.); 4 Anatomy and Cell Differentiation Lab, Department of Medicine and Aging Sciences, “G. D’Annunzio” University of Chieti—Pescara, 66100 Chieti, Italy; b.ghinassi@unich.it; 5 Cancer Science Institute, National University of Singapore, Singapore 117599, Singapore; 6 Department of Translational Medicine, University of Eastern Piedmont, 28100 Novara, Italy; andy.seelam@unimi.it (A.J.S.); mborchiellin@bidmc.harvard.edu (M.B.); 7 International Center for T1D, Pediatric Clinical Research Center “Romeo Ed Enrica Invernizzi”, Department of Biomedical and Clinical Science L. Sacco, Università Degli Studi Di Milano, 20157 Milan, Italy; 8 Division of Translational Genomics, Exploratory Oncology Research & Clinical Trial Center, National Cancer Center, Kashiwa 277-8577, Japan

**Keywords:** epigenetics, DNA methylation, NAD, gene regulation

## Abstract

Nutritional intake impacts the human epigenome by directing epigenetic pathways in normal cell development via as yet unknown molecular mechanisms. Consequently, imbalance in the nutritional intake is able to dysregulate the epigenetic profile and drive cells towards malignant transformation. Here we present a novel epigenetic effect of the essential nutrient, NAD. We demonstrate that impairment of DNMT1 enzymatic activity by NAD-promoted ADP-ribosylation leads to demethylation and transcriptional activation of the *CEBPA* gene, suggesting the existence of an unknown NAD-controlled region within the locus. In addition to the molecular events, NAD- treated cells exhibit significant morphological and phenotypical changes that correspond to myeloid differentiation. Collectively, these results delineate a novel role for NAD in cell differentiation, and indicate novel nutri-epigenetic strategies to regulate and control gene expression in human cells.

## 1. Introduction

Malnutrition and obesity are associated with epigenetic dysregulation, thereby promoting cellular transformation and cancer initiation [1,2]. A prolonged exposure to a high-fat diet, poor nutrition, and insults from environmental toxicants all contribute to the epigenetic transgenerational inheritance of obesity [3]. The degree of obesity, in terms of body weight, is a well-documented risk factor for hematopoietic disease and cancer [4,5]. Together, this evidence highlights the importance of balanced micronutrient intake in order to preserve cell-specific epigenetic programming and prevent anomalies that can potentially result in malignant transformation [6,7].

In the last decade, numerous studies have focused on establishing a link between nutrition and epigenetics. This led to the concept of “Precision Nutrition”: a translational approach based on the use of dietary compounds to direct epigenetic changes and drive normal cellular development [8]. Natural compounds, like vitamins C and D, have been shown to slow pathological processes through their impact on the epigenome [9,10]. Similarly, nutri-epigenomic approaches have been shown to prevent several disease conditions, including cancer [11,12]. Nevertheless, the molecular mechanisms by which nutrients modulate the epigenome of healthy or cancer cells is largely unknown.

Nicotinamide adenine dinucleotide (NAD) is a dietary compound essential for life, and a coenzyme implicated in cellular redox reactions [13]. Maintenance of adequate levels of NAD is critical for cellular function and genomic stability [14]. Few reports have shown that NAD precursors such as vitamin B3 (or nicotinic acid, NA) and nicotinamide (Nam) are able to drive cell differentiation in leukemic cell lines [15,16], and impair cell growth. However, the molecular mechanisms participating in these morphological changes remain unknown.

DNA methylation is a key epigenetic signature involved in transcriptional regulation, normal cellular development, and function [17]. Methyl groups are added to the carbon 5 of cytosines in the contest of CpG dinucleotides by specialized enzymes collectively known as DNA methyltransferase enzymes (DNMT1, 3A and 3B). While the bulk of the genome is methylated at 70–80% of its CpGs, CpG islands (CGI) that are clusters of CpG dinucleotides, generally proximal to the transcription start sites (TSSs) of most human protein-coding genes, are mostly unmethylated in somatic cells. Numerous studies have established a link between aberrant promoter DNA methylation and gene silencing in diseases such as cancer [18,19].

NAD is also the substrate of Poly-(ADP) Ribose Polymerase 1 (PARP1), a nuclear protein that plays a pivotal role in gene regulation and chromatin remodeling [20,21]. PARP1 utilizes NAD as a source of ADP-ribose moieties to assemble ADP-ribose polymers (PAR) and coordinate epigenetic modifications including DNA methylation [22,23]. Several experimental data support a PARP1-mediated inhibition of DNA methyltransferase 1 (DNMT1) activity in human cell lines [24,25]. These findings suggest a role for NAD in altering and/or facilitating modulation of DNA methylation, even if a direct link between demethylation and NAD treatment has not been established [26,27].

Herein, we present a novel function of NAD: the ability to specifically demethylate and induce the expression of the hematopoietic master regulator, the CCAAT/enhancer binding protein alpha (*CEBPA*) gene locus. The demethylation effect correlates with a total and local increase of ADP-ribose polymers (PAR) at the *CEBPA* promoter, thus supporting a NAD/PARP1/DNMT1 axis in which local inhibition of DNMT1 results in site-specific demethylation and transcriptional activation.

Our findings indicate NAD as a novel epigenetic modulator that counteracts the widespread epigenetic reprogramming contributing to obesity and cancers, and provides the first nutritional-based therapy for clinical interventions in these conditions.

## 2. Materials and Methods

### 2.1. Cells and Cell Culture

K562, HEK293 and Jurkat cell lines were purchased from ATCC. and were grown in RPMI (K562 and Jurkat) or DMEM (HEK293) medium supplemented with 10% fetal bovine serum (FBS), in the absence of antibiotics and both at 37 °C, 5% CO_2_. The K562-CEBPA-ER line was grown in a 12 well plate in phenol red–free RPMI 1640 (Thermo Fisher Scientific, Whaltman, MA, USA; Cat. No. 11835030), supplemented with 10% Charcoal stripped FBS (Sigma Aldrich, St. Louis, MO, USA; Cat. No. F6765), and 1 µg/mL puromycin, beginning at a density of 0.2 × 10^6^ cells/mL. 1 µM estradiol (Sigma Aldrich St. Louis, MO, USA; Cat. No. E2758) was added from a 5-mM stock solution in 100% ethanol to induce CEBPA-ER nuclear translocation and a corresponding amount of ethanol (0.02%) to mock-treated cells as controls. Viable cells excluding trypan blue were enumerated every day and used for the experiment [28,29].

### 2.2. NAD Treatment

K562 cells were incubated with 0.1, 0.5, 1, 1.5 or 10 mM of NAD (Sigma Aldrich St. Louis, MO, USA) or vehicle (milliQ water) for four days at 37 °C. Cells were counted every day and cell pellets were collected to perform all the downstream analysis. The NAD content was assessed by the BioVision NAD/NADH Quantification Colorimetric Kit according to the manufacturer’s instructions (BioVision, San Francisco, CA, USA). Briefly, K562 cells were homogenized by two cycles of freezing and thawing in 400 μL of BioVision NAD/NADH extraction buffer. The homogenate was filtered using BioVision 10-kD cut-off filters (10,000 g, 25 min, 4 °C). To detect only NADH content, NAD was decomposed by heating 200 µL of the homogenate. The homogenate of decomposed and non-decomposed samples was distributed in a 96 well plate, and the developer solutions was added to the samples. The absorbance (OD 450 nm) was acquired for 30 min using the VICTOR Multilabel Plate Reader (Perkin Elmer, Whaltman, MA, USA).

### 2.3. K562 Wright Giemsa Staining

Approximately 2 × 10^4^ per each sample, were spotted on a slide using the cytospin at 400 rpm for 5 min. The cells were then stained with the Wright Giemsa solutions kit (CAMCO STAIN PAK Fort Lauderdale, FL, USA, pc#702) according to manufacturer’s instructions.

### 2.4. Nitroblue Tetrazolium (NBT) Assay

Nitroblue blue tetrazolium (NBT) analysis was performed using 5 × 10^5^ cells incubated in a 1 mL solution containing phosphate-buffered saline (PBS), NBT (Sigma Aldrich, St. Louis, MO, USA), and 0.33 µM phorbol myristate acetate (PMA) for 20 min at 37 °C. The reaction was then stopped by incubation on ice. Cells were immediately fixed on slides by cytocentrifugation and counterstained with 0.5% safranin O in 20% ethanol.

### 2.5. Immunofluorescence

Cells were fixed with PFA 2% (Paraformaldehyde/MeOH), washed with 1X Phosphate-buffered saline (PBS), and permeabilized with 0.5% Triton X100. After blocking with 7% Goat-serum for 30 min, cells were incubated with primary antibody Anti poly (ADP-ribose) polymer (1:400 Abcam, Cambdrige, UK ab 14459) overnight at 4 °C, covered from the light. The following day, cells were washed with 1X PBS, and incubated with secondary antibody goat anti-mouse (1:500, Alexa Fluor 555) for 1 h, re-washed, and nuclei counterstained with Prolong gold Antifade Mountant already containing DAPI (Thermo Fisher Scientific, Whaltman, MA, USA). Samples were analyzed on a Leica DM 5500B Microscope with a 100 W high-pressure mercury lamp. Images were assembled and contrast-enhanced using Image J as per manufacturer’s recommendations.

### 2.6. RNA Isolation and qRT-PCR Analyses

Total RNA isolation was carried out using TRIzol (Thermo-Fisher Scientific, Whaltman, MA, USA), as previously described [23]. All RNA samples used in this study were treated with DNase I (10 U of DNase I per 3 µg of total RNA; 37 °C for 1 h; in the presence of RNase inhibitor). After DNase I treatment, RNA samples were extracted with acidic phenol (Sigma Aldrich, St. Louis, MO, USA; pH 4.3) to eliminate any remaining traces of DNA. Taqman based qRT-PCR was performed using the one step Affymetrix HotStart-IT qRT-PCR Master Mix Kit (Affymetrix USB, Whaltman, MA, USA) and 50 ng of total RNA per reaction. Amplification conditions were 50 °C (10 min), 95 °C (2 min), followed by 40 cycles of 95 °C (15 s) and 60 °C (1 min). Target gene amplification was calculated using the formula 2^−∆∆Ct^ as described [23], primer and probe sequences are listed in Supplementary Table S1.

### 2.7. DNA Isolation

Cell pellets, resuspended in a homemade lysis buffer (0.5% SDS, 25 mM EDTA pH 8, 10 mM TRIS pH 8, 200 mM NaCl), were initially treated with RNase A (Roche, Basel, Switzerland) for 20 min at 37 °C and then Proteinase K (Roche) overnight at 65 °C. High quality genomic DNA was extracted by Phenol:chloroform:isoamyl Alcohol 25:24:1, pH:8 (Sigma Aldrich, St. Louis, MO, USA) and precipitated with Isopropanol the following day. Genomic DNA was resuspended in Tris 1 mM, EDTA 10 mM (TE) pH 8 and stored at 4 °C.

### 2.8. Western Blotting Analysis

Whole-cell lysates from approximately 2 × 10^5^ cells per each sample were separated on 15% SDS-PAGE gels and transferred to a nitrocellulose membrane. Immunoblots were all blocked with 5% nonfat dry milk in Tris-buffered saline, 0.1% (TBS-T) prior to incubation with primary antibodies. The Anti-poly (ADP-ribose) polymer (1:1000 Abcam, ab14459) was stained overnight at 4 °C. For PARP1 and DNMT1 protein analyses, equivalent amount of whole-cell lysates were separated on 7% SDS-PAGE gels and transferred to a nitrocellulose membrane. Immunoblots were stained overnight with the following primary antibodies: Anti-PARP1 (1:1000 Active motif, Carlsbad, CA, USA; 39559), Anti-DNMT1 (1:1000, Abcam, Cambdrige, UK; ab19905). All secondary horseradish peroxidase (HRP)-conjugated antibodies were diluted 1:5000 and incubated for 1 h at room temperature with TBST/5% milk. Immuno-reactive proteins were detected using the Pierce^®^ ECL system (Thermo Scientific, Whaltman, MA, USA; #32106).

### 2.9. Bisulfite Sequencing and Analysis

DNA methylation profile of *CEBPA* locus was analyzed by bisulfite sequencing as previously described [27]. Briefly, high molecular weight genomic DNA (1 µg) was subjected to bisulfite conversion using the EZ DNA Methylation-Direct kit (Zymo Research, Irvine, CA, USA) following the manufacturer’s instructions. Polymerase chain reactions (PCR) on bisulfite converted DNA was performed with FastStart Taq DNA Polymerase (Roche, Basel, Switzerland) with the following conditions: 95 °C (6 min) followed by 35 cycles at 95 °C (30 s) 53–57 °C (1 min) 72 °C (1 min), and a final step at 72 °C (7 min). Primers and PCR conditions for bisulfite sequencing are summarized in Supplementary Table S2. After gel purification, the amplicon was cloned into pGEM T-easy vector (Promega, Madison, WI, USA) and the plasmid transformed in E. coli competent cells JM109 (Promega, Madison, WI, USA). Nine positive clones were analyzed by Sanger sequencing for each sample. Only clones with a conversion efficiency of at least 99.6% were considered for further processing by QUMA: a quantification tool for methylation analysis (http://quma.cdb.riken.jp/ (accessed on 24 March 2021)) [30].

### 2.10. Chromatin Immunoprecipitation

ChIP was performed as previously described [31]. Briefly, K562 cells were crosslinked with 1% formaldehyde (formaldehyde solution, freshly made: 50 mM HEPES-KOH; 100 mM NaCl; 1 mM EDTA; 0.5 mM EGTA; 11% formaldehyde) for 10 min at room temperature (RT) and 1/10th volume of 2.66 M glycine was then added to stop the reaction. Cell pellets were washed twice with ice-cold 1X PBS (freshly supplemented with 1 mM PMSF). Pellets of 2 × 10^6^ cells were used for immunoprecipitation and lysed for 10 min on ice and chromatin fragmented using a Bioruptor Standard (30 cycles, 30 s on, 60 s off, high power). Each ChIP was performed with 10 µg of antibody, incubated overnight at 4 °C. A slurry of protein A or G magnetic beads (NEB, Ipswich, MA, USA) was used to capture enriched chromatin, which was then washed before reverse-crosslinking and proteinase K digestion at 65 °C. Beads were then removed in the magnetic field and RNase treatment (5 µg/µL Epicentre, Charlotte, NC, USA; MRNA092) was performed for 30 min at 37 °C. ChIP DNA was extracted with Phenol:chloroform:isoamyl Alcohol 25:24:1, pH 8 (Sigma Aldrich, St. Louis, MO, USA) and then precipitated with equal volume of isopropanol in presence of glycogen. DNA pellet was dissolved in 30 µL of TE buffer for following qPCR analyses. The following antibodies were used for ChIP: Anti-DNMT1 (Abcam, Cambdrige, UK; ab19905), Anti-poly (ADP-ribose) polymer (Abcam, Cambdrige, UK; ab14459), normal mouse IgG (Millipore, Burlington, MA, USA; 12-371b) and normal rabbit IgG (Cell Signaling, Danvers, MA, USA; 2729S). Fold enrichment was calculated using the formula 2 ^−ΔΔCt^ (ChIP/non-immune serum)). Primer sets used for ChIP are listed in Supplementary Table S3.

### 2.11. Immunostaining for FACS Analysis

Anti-CD15-APC (Thermo Fisher Scientific, Whaltman, MA, USA; Cat. No. 17-0158-42), anti-CD14-FITC (Thermo Fisher Scientific, Whaltman, MA, USA; Cat. No. 11-0149-42) and anti-CD11b-Pacific blue (BioLegend, San Diego, CA, USA; Cat. No. 101224) were incubated with 1 × 10^6^ K562 cells (vehicle or NAD treated) at 1:100 ratio. Cells were pre-incubated with anti-Fc receptor antibody (Thermo Fisher Scientific, Whaltman, MA, USA Cat. No. 14-9161-73) at 1:20 ratio to block Fc receptor before staining. Zombie red staining (BioLegend, San Diego, CA, USA Cat. No. 423109) was used as cell viability dye during FACS analysis. Cells were fixed using 2% PFA (Sigma Aldrich, St. Louis, MO, USA; Cat. No. 158127) before performing FACS analysis. Cell acquisition and analysis were performed on BD LSRFortessa (BD Biosciences, Franklin Lakes, NJ, USA) using BD FACSDivaTM software (BD Bioscience, Franklin Lakes, NJ, USA). Analysis was performed using Flowjo software (Flowjo LLC, Ashland, OR, USA).

### 2.12. Flow Cytometry Analysis for Cell Cycle Distribution

Approximately 1 × 10^6^ cells were resuspended in 500 μL cold Hypotonic solution containing 50 μg/mL PI (Sigma Aldrich, St. Louis, MO, USA), 0.1% Triton X-100 (Merck Millipore, Burlington, MA, USA), 100 μg/mL RNase (Sigma Aldrich, St. Louis, MO, USA) and 0.1% sodium citrate solution. Tubes were placed in the dark at 4 °C. Analysis was made between 20 min and 2 h of incubation by flow cytometry. Cell acquisition was performed on BD LSRFortessa (BD biosciences, Franklin Lakes, NJ, USA) using BD FACSDivaTM software (BD Bioscience Franklin Lakes, NJ, USA) and analysis using Flowjo software (Flowjo LLC, Ashland, OR, USA).

### 2.13. Annexin V Staining

An FITC Annexin V Apoptosis Detection Kit I (BD Bioscience Franklin Lakes, NJ) was used to determine the percentage of K562 undergoing apoptosis upon NAD treatment. All samples were prepared following the manufacturer’s instructions. Briefly, cells were collected every day, washed twice with cold PBS, and then resuspended in 1×Xbinding buffer at a concentration of 1 × 10^6^ cells/mL.

Cells were incubated with 5 µL fluorescein isothiocyanate (FITC) annexin V and 5µL Propidium Iodide for 15 min at room temperature in darkness. Analyses of cells viability and apoptosis were performed on BD LSR Fortessa (BD biosciences, Franklin Lakes, NJ, USA) using BD FACSDivaTM software (BD Bioscience Franklin Lakes, NJ). The data analysis was performed using Flowjo software (Flowjo LLC, Ashland, OR, USA).

### 2.14. Seahorse Analysis

A Mito Stress Test (Agilent Seahorse, Agilent, Santa Clara, CA, USA; 103015-100) assay was run as per manufacturers’ recommendations. Briefly, on the day of assay, counted and PBS washed cells were suspended in XF Assay media (Agilent Seahorse Bioscience), pH adjusted to 7.4 ± 0.1, supplemented with 4.5 g/L glucose (Sigma-Aldrich, St. Louis, MO, USA; G7528), 0.11 g/L sodium pyruvate (Sigma-Aldrich, St. Louis, MO, USA) and 8 mM L-glutamine (Sigma-Aldrich, St. Louis, MO, USA). 1 × 10^5^ cells were added to each well of XFe24 Cell-Tak (Corning, New York, NY, USA) pre-coated culture plates and then slowly centrifuged for incubation at 37 °C in a non-CO^2^ incubator. Oxygen consumption rate was measured at baseline using a Seahorse XFe24 according to standard protocols and after the addition of oligomycin (100 μM), carbonyl cyanide-4-(trifluoromethoxy) phenylhydrazone (FCCP, 100 μM), rotenone, and antimycin A (50 μM). Fold change was determined by normalizing raw values to the average of the second basal reading.

### 2.15. Statistical Analysis

All bisulfite sequenced clones were analyzed by Fisher’s exact test. For mRNA qRT-PCR, *p*-values were calculated by *t*-test in GraphPad Prism Software. For both the analysis, values of *p* < 0.05 were considered statistically significant (* *p* < 0.05; ** *p* < 0.01; *** *p* < 0.001). The mean ± SD of duplicates is reported.

## 3. Results

### 3.1. NAD Inhibits Cancer Cell Growth in a Dose-Dependent Manner and Drives Accumulation of Intracellular Poly ADP-Ribose Polymers

**Figure 1:**
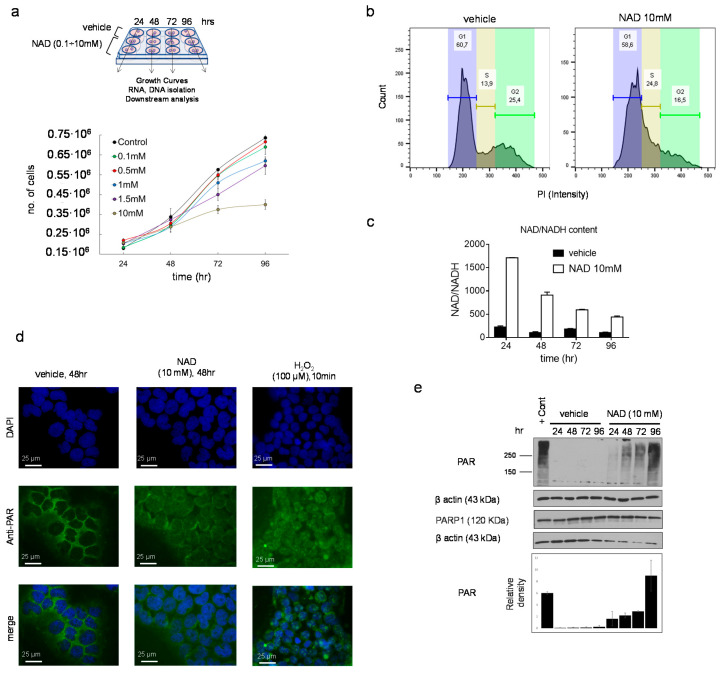
**NAD inhibits cancer cell growth in a dose-dependent manner and drives accumulation of intracellular poly ADP-ribose polymers**. (**a**, **upper panel**) Schematic of the experiment. K562 cells were cultured at different concentration of NAD: 0.1, 0.5, 1, 1.5, 10 mM or vehicle. Cell pellets, RNA, and DNA samples were collected at different time points: 24, 48, 72, 96 h. (**a**, **lower panel**) K562 growth curves in presence of NAD or vehicle. Cells were counted every 24 h for four days. The error bars represent the mean ± standard deviation of four independent experiments (*n* = 4). (**b**) Propidium iodide staining of K562 grown in presence of NAD (10 mM) for 72 h and cell cycle analysis by FACS showing a decrease in cells in G2 phase, from 25% to 16%, indicating arrest in the cell division rate. (**c**) The NAD/NADH content measured by colorimetric assay. The absorbance was measured at 450 nm every 24 h from the addition of NAD (10 mM) to the cell culture media. The NAD ratio was calculated according to the manufacturer’s instructions (BioVision). (**d**) Immunofluorescence of PARs in K562 supplemented with NAD (10 mM) or vehicle after 24 h. A positive control is shown in the right column and was obtained growing K562 in presence of 100 µM of H_2_O_2_ for 10 min. (**e**) PAR and PARP1 protein levels in K562 cells treated with NAD. The immunoblot band densities is measured using ImageJ and normalized by β-Actin.

NAD precursors drive myeloid differentiation and impair cell growth [15,16]. To examine whether similar effects could be mediated by NAD, K562 cells were cultured following a single addition of NAD or vehicle to the media, and tracked over four days (Figure 1a). Cells were counted every day and cell pellets collected for downstream analyses (Figure 1a). Inhibition of the cell growth was observed across all the tested NAD concentrations in a dose-dependent manner, with the strongest effect at 10 mM, 96 h upon treatment (Figure 1a). Notably, this inhibition was not associated with apoptosis as demonstrated by the Annexin V staining, thus showing high viability (≈85%) of NAD-treated cells versus untreated (Figure S1a). Cell cycle analysis performed at 72 h after treatment revealed a decrease of cells in G2 phase, from 25% to 16%, indicating an arrest in the cell division rate (Figure 1b). Consistently, the NAD/NADH content in the 10mM NAD treated cells displayed a nearly eightfold increase as compared to the baseline 24 h after treatment (Figure 1c). Provided that NAD is partially utilized as a source of ADP-ribose units by PARP1 to build linear and branched poly ADP-ribose (PAR) polymers, NAD-treated and untreated K562 were stained with an anti-PAR antibody and examined by immunofluorescence to monitor the accumulation of PAR. As expected, 24 h upon NAD treatment, cells displayed an intense fluorescence signal in treated as compared to untreated cells, caused by the increase in PAR synthesis and accumulation (Figure 1d). These results mirrored the effects induced by 10-min treatment with hydrogen peroxide (H_2_O_2_), a known DNA damaging agent [32,33,34] associated with PAR production and therefore used as a positive control (Figure 1d). Overall, these findings supported PAR accumulation driven by NAD. As a further validation, PAR levels were analyzed by western blot. The strongest PAR band was detected on the first day and then gradually decreased in the following days (Figure 1e), likely due to PAR’s degradation by poly (ADP-ribose) glycohydrolases (PARGs) or similar pathway-related enzymes [35].

Collectively, these data demonstrate that NAD inhibits cell growth and mediates accumulation of intracellular PAR as early as 24 h after treatment.

### 3.2. NAD Treatment Induces CEBPA Distal Promoter Demethylation

**Figure 2:**
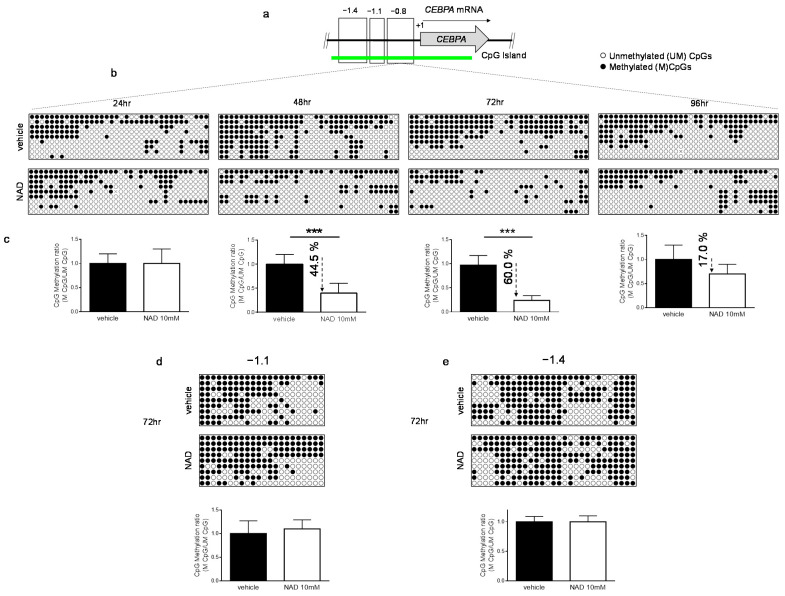
**DNA methylation patterns of *CEBPA* upon NAD (10 mM) or vehicle treatment.** (**a**) Schematic representation of *CEBPA* locus. The three regions analyzed in the promoter of *CEBPA* located at −0.8 kb (−557; −857), −1.1 kb (−895; −1.122) or −1.4 kb (−1.120; −1.473) from the TSS (+1) of the gene. (**b**,**c**) The methylation status of the distal promoter (the −0.8 kb region) was assessed at the four indicated time points (*n* = 9 clones). Lollipop graphs were generated using QUMA software. CpG methylation ratio, consisting of methylated CpGs divided by unmethylated CpGs, was calculated by QUMA software. (**d**,**e**) Methylation status of distal promoter I (−1.1 kb) and distal promoter II (−1.4 kb) 72 h upon NAD (10 mM) addition. Lollipop graphs were generated as described (*n* = 9 clones). All bisulfite sequenced clones were analyzed by Fisher’s exact test, ***: *p* < 0.001.

A PARP1-mediated inhibition of DNMT1 activity in human cell lines has been reported [23,24]. Therefore, we reasoned that an increase of NAD, a substrate of PARP1, could modulate genomic methylation. To this end, we investigated the methylation dynamics of the well-studied methylation-sensitive gene *CEBPA* in K562 cells, following treatment with 10 mM NAD [31,36]. CEBPA is a master transcription factor in the hematopoietic system, the loss or inhibition of which can result in a block of differentiation and granulopoieisis, contributing to leukemic transformation. The *CEBPA* promoter is aberrantly methylated in ~30% and ~51% of patients with chronic myeloid leukemia and acute myeloid leukemia, respectively [16,36,37]. The promoter, encompassing the −1.4 kb to −0.5 kb regions distal to the transcriptional start site (TSS), is heavily methylated in K562. Thus, we decided to assess the impact of NAD treatment on a DNA methylation profile. Using bisulfite sequencing, we surveyed three distinct regions located at −0.8 kb (−557; −857), −1.1kb (−895; −1.122), −1.4 kb (−1.120; −1.473) upstream to the TSS of *CEBPA* (Figure 2a). NAD treatment led to concomitant decrease in DNA methylation levels within the distal promoter region (−0.8 kb) (Figure 2b,c and Figure S2a) which equaled a 44% reduction at 48 h and 60% at 72 h after NAD addition. These levels bounced back to a mild 17% and then decreased after 96 h, suggesting a dynamic re-establishment of DNA methylation levels within the site (Figure 2b,c). A positive control using H_2_O_2_ to demethylate the distal promoter is shown in the Supplementary Materials (Figure S1b). Consistent with our earlier findings, wherein the strongest accumulation of PARs was observed 24 h post-NAD treatment (Figure 1d,e), these results seem to indicate that the additional 24 h were required to inhibit DNMT1 enzymatic activity and promote the methylation changes observed over the 48 and 72 h time points (Figure 2b,c). Unexpectedly, only minor changes in the distal promoter I (−1.1 kb) and II (−1.4 kb) were detected at 72 h, suggesting a certain specific modality of NAD-mediated demethylation (Figure 2d,e). As previously reported, DNA methylation within the −1.1 kb and −1.4 kb regions does not correlate with *CEBPA* expression in both K562 and AML samples using conventional hypomethylating drugs [36].

Together these data demonstrate that NAD-induced *CEBPA* promoter demethylation relies on a PAR-dependent mechanism which impairs DNMT1 activity.

### 3.3. NAD Treatment Enhances CEBPA mRNA Transcription in K562 by a PARP1-Dependent Mechanism

**Figure 3:**
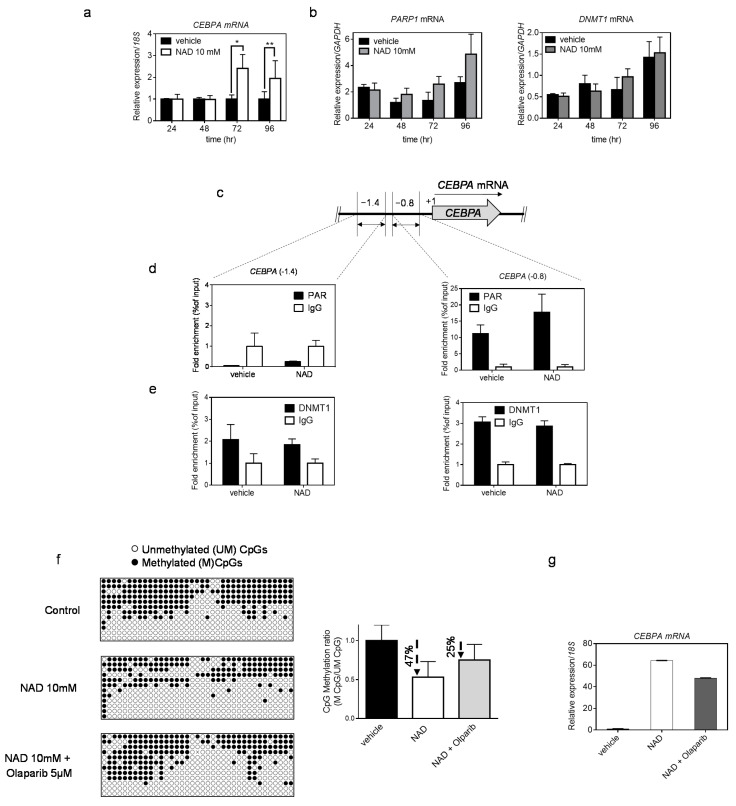
**NAD treatment enhances *CEBPA* transcription in K562 by a PARP1-dependent mechanism**. Panel (**a**) shows *CEBPA* mRNA levels after four days of treatment with NAD. qRT–PCR bars represent the mean ± s.d. of four independent experiments (*n* = 4). Panel (**b**) shows PARP1 and DNMT1 mRNA levels after four days of treatment with NAD. qRT–PCR bars represent the mean ± s.d. of three independent experiments(*n* = 3). Chromatin was collected to perform ChIP assays with antibodies to PAR, DNMT1 and IgG (**c**–**e**). (**c**) Schematic of the *CEBPA* promoter regions screened by ChIP-qPCR analysis respectively at −1.4 kb and −0.8 kb from the TSS (double-headed arrows). (**d**) ChIP using PAR antibody and qPCR analysis of regions −1.4 kb (left panel) and −0.8 kb (right panel). (**e**) ChIP using DNMT1 antibody and qPCR analysis of regions −1.4 kb (left panel) and −0.8 kb (right panel). Error bars indicate ± S.D. *: *p* < 0.05; **: *p* < 0.01. (**f**,**g**) K562 cells were cultured in presence of + NAD 10 mM alone, Olaparib 5 µM + NAD 10mM or vehicle. The methylation status of the distal promoter (−0.8 kb region) after treatment was assessed at the third day (*n* = 9 clones). Lollipop graphs were generated using QUMA software. CpG methylation ratio, consisting of methylated CpGs divided by unmethylated CpGs, was calculated by QUMA software. (**g**) *CEBPA* mRNA levels upon NAD treatment. qRT–PCR bars indicate mean ± s.d. of three independent experiments (*n* = 3).

DNA methylation is a key epigenetic signature involved in gene regulation. To investigate whether NAD-induced demethylation of the *CEBPA* distal promoter was associated with increased levels of *CEBPA* transcriptional activation, we measured the *CEBPA* expression by qRT PCR in cells treated with 10 mM NAD (Figure 3a) over multiple time points. Upregulation of *CEBPA* 72- and 96-h after treatment was observed only in cells treated with the highest NAD concentration (Figure 3a and Figure S2a); a positive control is shown in the Supplementary Materials (Figure S2b). These results parallel *CEBPA* upregulation at 72- and 96-h following demethylation of the distal promoter using the standard hypomethylating agent 5-aza-2′-deoxycytidine in K562 cells [36]. As the only region sensitive to the NAD-induced demethylation effect corresponded to the *CEBPA* distal promoter, while nearly no changes occurred in the two upstream regions (−1.4 kb and −1.1kb), we reasoned that the involvement of epigenetic regulators accounted for this site selectivity. Previous studies have reported that PARP1 assembled ADP-ribose polymers are able to impair DNMT1 activity in human and murine cell lines [23]. In following these findings, we hypothesized a mechanism wherein the NAD-induced production of PAR would specifically inhibit DNMT1 activity at the *CEBPA* distal promoter, without affecting the more upstream regions. To test this hypothesis, we firstly verified that the levels of PARP1 and DNMT1 were not influenced by NAD at both the expression and protein levels (Figure 3b and Figure S2c). Secondly, we performed quantitative Chromatin Immunoprecipitation with anti-PAR and anti-DNMT1 antibodies 24 h after NAD treatment (Figure 3c–e), given the strongest increase of PAR polymers at that specific time point (Figure 1d,e). As expected, the *CEBPA* distal promoter region exhibited more than a 1.6-fold enrichment of PAR polymers than did the vehicle treated cells. In the distal promoter II (Figure 3d,e), the polymers were absent. Interestingly, DNMT1 distribution between the distal promoter and the regions more upstream was unchanged (Figure 3e). This suggests the same accessibility of DNMT1 for both sites, and a potential impairment of the enzymatic activity at the distal promoter due to the presence of the PAR polymers.

Collectively, these results indicate a PARP1-dependent demethylating mechanism boosted by NAD levels, and an enabling inhibition of DNMT1 activity in selected loci.

In order to further validate the hypothesis of a PARP1-dependent demethylating mechanism, a pharmacological inhibition of PARP1 with Olaparib [38,39] was performed in addition to the NAD treatment. Cell growth was monitored using several concentrations of Olaparib, as previously reported [39] (Figure S2d), and the 5 µM concentration was chosen to treat K562 cells in combination with NAD 10mM. Methylation levels of the *CEBPA* distal promoter and *CEBPA* mRNA expression were assessed at 72 h after the NAD “only” or NAD and Olaparib treatment (Figure 3f,g). The percentage of demethylation reached 47% using NAD only, while the addition of Olaparib led to a drop of 25% (Figure 3f). The same trend was observed for *CEBPA* expression. Reduced *CEBPA* activation was detected in cells treated with both NAD and Olaparib (Figure 3g)

**Figure 4:**
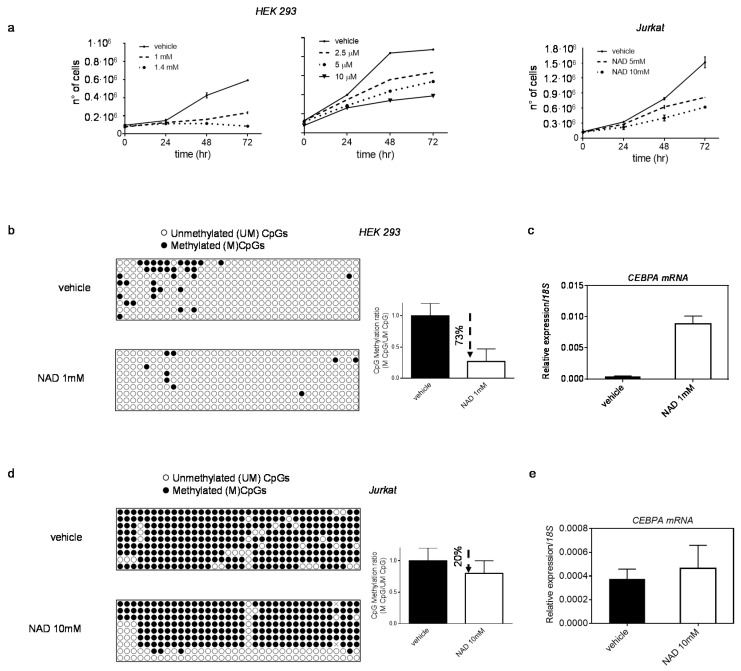
**Effect of NAD treatment on *CEBPA* transcription in HEK 293 and Jurkat**. Panel (**a**) HEK 293 and Jurkat growth curves in presence of NAD or vehicle. Cells were counted every 24 h for four days (*n* = 3). (**b**,**c**) DNA methylation changes of *CEBPA* distal promoter (the −0.8 kb region) in HEK 293 (**b**), and corresponding increase in *CEBPA* mRNA levels (**c**) 72 h upon treatment with NAD. (**d**,**e**) DNA methylation changes of *CEBPA* distal promoter (the −0.8 kb region) in Jurkat (**d**), and corresponding increase in *CEBPA* mRNA levels (**e**), 72 h upon treatment with NAD. Lollipop graphs were generated using QUMA software. CpG methylation ratio, consisting of methylated CpGs divided by unmethylated CpGs, was calculated by QUMA software in all clones analysed per each construct (*n* = 9). qRT–PCR, bars indicate mean ± s.d of three independent experiments (*n* = 3).

Finally, to rule out a cell line-dependent effect, we verified the NAD-induced demethylation of the *CEPBA* distal promoter and consequent *CEBPA* transcription activation in two other cell lines: HEK 293 (human embryonic kidney) and Jurkat (human T-lymphocyte), using optimized NAD concentrations (Figure 4).

In both cell lines, the CEPBA locus is methylated to a various extent, and *CEBPA* expression is low or undetectable (Figure 4b–e). Upon treatment, the methylation profile of *CEBPA* locus and expression levels of mRNA were investigated. Reduction in DNA methylation levels was observed in HEK293, which showed the strongest drop (~73%), followed by a remarkable *CEBPA* reactivation (nearly 96% increase) (Figure 4b,c). However, no changes in *CEBPA* transcription were detected in Jurkat, where the degree of demethylation was considerably lower (20% reduction) than HEK 293 72 h after treatment (Figure 4d,e).

These results suggest that the NAD-induced demethylation is not restricted to a specific cell line but occurs broadly in other systems as well.

### 3.4. NAD Induces Myeloid Differentiation

**Figure 5:**
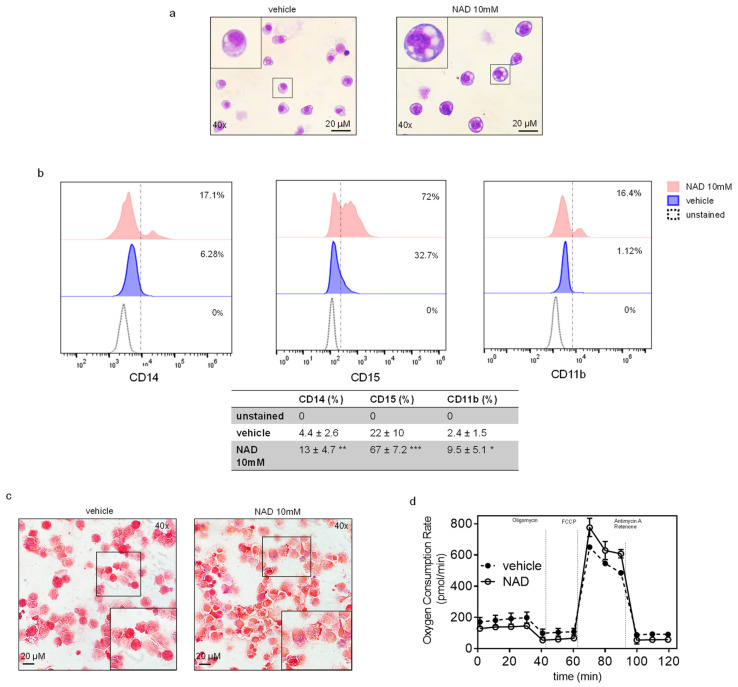
**NAD induces myeloid differentiation in K562.** (**a**) Wright Giemsa staining showing morphological changes between NAD-treated and control cells after four days. The figure is representative of five independent experiments (**b**) Representative image showing the increase in the surface markers CD15, CD14 and CD11b upon NAD treatment by flow cytometry. The table reports the mean % ±SD of CD14^+^, CD15^+^, CD11b^+^ cells of five independent experiments. *: *p* < 0.05; **: *p* < 0.01; ***: *p* < 0.001 vs. vehicle (*n* = 5) (**c**) NBT positive staining detected by small blue dots after counterstaining the cells with safranin. A magnification is shown in the rectangle. The pictures are representative of five independent experiments (**d**) Seahorse XF analysis of K562 mitochondrial stress response in cells treated with NAD or vehicle. The figure represents the mean of three biological replicates (*n* = 3). Error bars indicate ± s.d.

As previously reported, NAD-precursors such as NA and other niacin-related compounds induce differentiation in immortalized cell lines, such as K562 and HL60 [15,16]. These findings prompted us to assess morphological changes upon NAD treatment. Wright Giemsa staining of K562 treated with 10 mM NAD or vehicle revealed striking morphological changes four days after treatment (Figure 5a). Specifically, vehicle-treated cells exhibited a homogeneous population of round-shaped cells, with round or oval cell nuclei, whereas NAD-treated cells were more heterogeneous, with a higher cytoplasm:nucleus ratio, eccentrically located reniform nuclei with dense regions of heterochromatin, and numerous vacuoles resembling a monocytic-macrophagic morphology. Additionally, NAD treatment leads to increases in nitroblue tetrazolium (NBT)-positive cells and expression of CD15, CD11b and CD14 surface markers, indicating that NAD promotesmonocytic-macrophagic differentiation in K562, (Figure 5b,c) [40]. Hence, despite the reactivation of *CEBPA* mRNA, which is a master regulator of granulocytic differentiation, the expected morphological changes were not detected in NAD treated cells, although we could confirm increased expression of both CD15 and CD11b and not CD14 upon ectopic expression of CEBPA protein as shown previously [40,41] (Figure S2e). These results are unsurprising since the oncogenic fusion protein BCR-ABL, which is constitutively expressed in K562, suppresses CEBPA translation. This suppression leads to transcriptional suppression of the granulocyte colony-stimulating factor receptor G-CSF-R and other myeloid precursor cells critical for granulocytic differentiation [41]. Along with these data, we confirmed the absence of CEBPA protein by western blot analysis on K562 NAD-treated cells (data not shown).

### 3.5. NAD Treatment Improves Mitochondrial OXPHOS Function

NAD has been previously demonstrated to restore mitochondrial function in aged mice and to increase the intracellular ratio of NAD+/NADH, a critical cellular balance required for sirtuin 1 (SIRT1) mediated activation of mitochondrial biogenesis [42,43]. To further investigate the NAD contribution to the mitochondrial function of K562, the Mito Stress Test was performed using a Seahorse XFe24 (Figure 5d). Basal oxygen consumption rate (OCR) was used as a surrogate measure of mitochondrial function since mitochondria utilize oxygen to generate mitochondrial ATP. Our results showed that NAD-treated K562 cells displayed a marginal increase in maximal oxygen consumption in response to Carbonyl cyanide-4 (trifluoromethoxy) phenylhydrazone (FCCP) stress. This translated to a 1.3-fold improvement in normalized maximal reserve capacity after only four days of co-incubation with NAD. Although the change in maximal reserve capacity post NAD-treatment was marginal, these results still highlight the significance that NAD treatment plays in improving mitochondrial health and perhaps contributing to the changes described. The entire profile of NAD-treated K562 does not depart drastically from untreated K562, but the increment of ORC emerging after the injection of FCCP indicates variations in respiration capacity of NAD-treated K562 versus untreated, subject to the same mitochondrial stimuli.

## 4. Discussion

**Figure 6:**
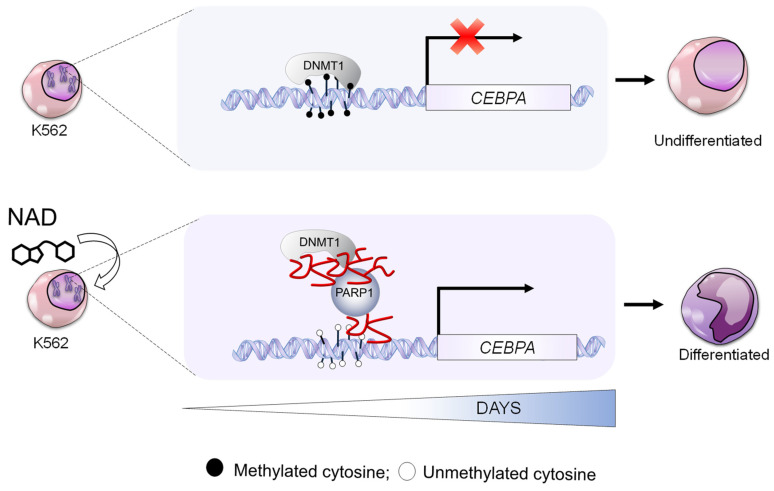
**Model showing the molecular mechanism of *CEBPA* gene reactivation by NAD**. *CEBPA* is epigenetically silenced in K562. DNMT1 ensure a constant methylated status of *CEBPA* promoter (upper panel). The NAD supplementation to K562 cell culture, boosts PARP1 to produce ADP-ribose polymers leading to DNMT1 inhibition (bottom panel). The ultimate effect is reactivated transcription of *CEPBA*.

This study explored the demethylation impact brought about by NAD treatment. On the example of the *CEBPA* gene locus, silenced by DNA methylation in the leukemia model used herein, we carried out a molecular and biological dissection of the potential mechanism implicated in NAD-induced demethylation. We demonstrated that impairment of DNMT1 enzymatic activity as a result of NAD-promoted ADP-ribosylation leads to the loss of *CEBPA* promoter methylation and corresponds to the transcriptional activation of *CEBPA* mRNA, thereby revealing an unknown NAD-controlled region within the *CEBPA* locus. The model describing the molecular mechanism is shown in Figure 6. Future studies will allow us to determine whether there are different levels of affinity of poly(ADP-ribose) for DNMT1 and for DNMT3a/DNMT3b and respective impairment of DNMT3a/DNMT3b enzymatic activity by NAD-promoted ADP-ribosylation

NAD is regarded as a potential antiaging molecule, the levels of which tend to decline over our lifetime. However, the molecular mechanisms linking low NAD levels to aging are only partially understood [44,45]. As a critical substrate of SIRT and PARP enzyme family members, which are involved in multiple epigenetic pathways (i.e., acetylation, ADP-ribosylation and DNA methylation), fluctuations of NAD levels may alter chromatin remodeling [43,46]. An additional epigenetic role for NAD, independently of its partnering enzymes, has been hypothesized by few reports wherein age- or nutrition-related decline of NAD levels were associated with the acquisition of abnormal DNA methylation profiles at specific loci [47,48]. In vitro evidence has also shown that ADP-ribosyl polymers impair DNMT1 enzymatic activity [23], and an ADP-ribosylation transcriptional control for the P16 and TET1 genes has been demonstrated [25,26]. To date, over 2300 proteins, including DNMT1, have been reported as ADP-ribosylated (http://ADPriboDB.leunglab.org (accessed on 24 March 2021)). How ADP-ribosylation preserves the unmethylated state of certain regulatory sequences remains elusive [49]. In every instance studied, we demonstrate that NAD treatment induces production of PAR polymers, site-specific demethylation of the *CEBPA* distal promoter, and results in transcriptional activation of *CEBPA* mRNA in K562 cells and HEK293 (Figure 2, Figure 3 and Figure 4). The moderate *CEBPA* activation in Jurkat (a T-cell line highly methylated in the *CEBPA* locus) is consistent with the considerably lower degree of demethylation (20% reduction) 72 h after NAD treatment, and suggests that prolonged timepoints might be needed to achieve demethylation levels resulting in mRNA expression. As a matter of fact, well-studied differentiation-inducing agents such as all-trans retinoic acid (ATRA) normally require a week if not longer to generate morphological changes [50,51]. Furthermore, the cell-type specific methylome could be discordantly perturbated by NAD administration, conferring a cell-type dependent response to the treatment. Future studies will clarify these mechanistic aspects.

In summary, all of our results led to our hypothes of a site-selective demethylation mechanism wherein the NAD-induced production of PAR polymers inhibits DNMT1 activity at the *CEBPA* distal promoter by preventing DNMT1 interaction with the CGI, as described in the depicted model (Figure 5). The co-occurrence of PARs and DNMT1 on the distal promoter, but not on the distal promoter I and II, suggests a PAR-mediated specific inhibition of DNMT1 and reveals a NAD-responsive element on the *CEBPA* promoter (Figure 3). Intriguingly, the morphological changes along with the pronounced NBT staining and the positive shift of CD15, CD11b and CD14 surface markers, in addition to the improved mitochondrial function, seem to point to a monocytic-macrophagic-like transcriptional activation program initiated by NAD treatment (Figure 5).

In conclusion, this study bridges a nutritional intervention to a molecular observation: an increase of NAD levels in a cancer cell line results in local correction of DNA methylation. These data, therefore, provide a nutritional-guided approach for the prevention and clinical management of cancers or other conditions associated with alteration of DNA methylation potentially linked to decreased NAD levels.

## Data Availability

Not applicable.

## Supplementary Materials

The following are available online at https://www.mdpi.com/article/10.3390/cells10112986/s1, Table S1: qRT-PCR primer sets; Table S2: Bisulfite primer sets; Table S3: ChIP-qPCR primer sets.
