# Supplementary material for: NAD Modulates DNA Methylation and Cell Differentiation
Source: Cells. 2021 Nov 2;10(11):2986. doi: 10.3390/cells10112986 (PMC8616462; doi:10.3390/cells10112986)
Supplement: Supplementary file 1 [file cells-10-02986-s001.zip › cells-1412127-supplementary.pdf]

Supplementary material

Table S1. qRT-PCR primer sets.

Primers	CEBPA
Forward	5'-TCGGTGGACAAGAACAG-3'
Reverse	5'-GCAGGCGGTCATTG-3'
Probe	5'-ACAAGGCCAAGCAGCGC-3'
	PARP1
Forward	5'-AAGATGATCTTTGATGTGGAAAGTA-3'
Reverse	5'-TGCCCTTGGGGAAGCTGAGCAAA-3'
Probe	5'-GAAGAAAGCCATGGTGGAGT-3'

DNMT1(ThermoFisher cat. No. hs00945875_m1); GAPDH (ThermoFisher cat. No. 4310884E); 18S rRNA (ThermoFisher cat.No. 4310893E).

Table S2. Bisulfite sequencing primer sets.

Region	Forward Primer	Reverse Primer
-0.8 (-557; -857)	5'-CAGCTCCGCTAGTCTGGGGGGCC-3'	5'-CACAGGGGTAGCCTGGAGATCAGA-3'
-1.1 (-895; -1.122)	5'-CACTCAAGGGGCCCCAGG-3'	5'-CCAGAGTTAAGTTTGTCTCC-3'
-1.4 (-1.120; -1.473)	5'-GGTGTTTTTAGCTGTGCCCCCT-3'	5'-TCAAGGGGCCCCAGGGCCT-3'

Table S3. ChIP-qPCR primer sets.

Region	Forward Primer	Reverse Primer
-0.8 (-557; -857)	5'-CAGCTCCGCTAGTCTGGGGGGCC-3'	5'-CACAGGGGTAGCCTGGAGATCAGA-3'
-1.4 (-1.120; -1.473)	5'-GGTGTTTTTAGCTGTGCCCCCT-3'	5'-TCAAGGGGCCCCAGGGCCT-3'
